# Metabolomic analyses of the bio-corona formed on TiO_2_ nanoparticles incubated with plant leaf tissues

**DOI:** 10.1186/s12951-020-00592-8

**Published:** 2020-02-17

**Authors:** Jasmina Kurepa, Timothy E. Shull, Jan A. Smalle

**Affiliations:** Plant Physiology, Biochemistry, Molecular Biology Program, Department of Plant and Soil Sciences, University of Kentucky, Lexington, KY 40546 USA

**Keywords:** Titanium dioxide nanoparticles, Flavonoids, Arabidopsis, *Transparent testa* (*tt*) mutants, Lipids

## Abstract

**Background:**

The surface of a nanoparticle adsorbs molecules from its surroundings with a specific affinity determined by the chemical and physical properties of the nanomaterial. When a nanoparticle is exposed to a biological system, the adsorbed molecules form a dynamic and specific surface layer called a bio-corona. The present study aimed to identify the metabolites that form the bio-corona around anatase TiO_2_ nanoparticles incubated with leaves of the model plant *Arabidopsis thaliana*.

**Results:**

We used an untargeted metabolomics approach and compared the metabolites isolated from wild-type plants with plants deficient in a class of polyphenolic compounds called flavonoids.

**Conclusions:**

These analyses showed that TiO_2_ nanoparticle coronas are enriched for flavonoids and lipids and that these metabolite classes compete with each other for binding the nanoparticle surface.

## Background

Titanium dioxide nanoparticles (TiO_2_ NPs) are widely used in food and cosmetics [1–3]. In addition to their use in sunscreens, paints, ointments, toothpaste—to name just some products—continuing efforts in the synthesis and modifications of TiO_2_ NPs have brought about new applications, most important being photovoltaics and remediation [1]. This increased use of TiO_2_ NPs also intensified studies of the environmental impact of this nanomaterial. Although TiO_2_ NPs were previously viewed as inert to the extent that they were used as a negative control in toxicological studies, sufficient evidence has recently accumulated indicating that exposure to TiO_2_ NPs impacts all organisms from bacteria to plants and humans [4–7]. In plants, most of the evidence showing that TiO_2_ NPs have an impact on whole-plant physiology has been gathered in crop species [8–10]. On the other hand, most of the cellular and molecular studies analyzing different aspects of TiO_2_ NP toxicity have been conducted in *Arabidopsis thaliana* due to the abundance of research resources available for this dicot model system [11]. Cellular studies in *A. thaliana* have shown that ultra-small TiO_2_ NPs can transverse cell walls and accumulate in vacuoles and nuclei [12]. Dependent on the exposure dose and time, ultra-small TiO_2_ NPs can disrupt microtubular networks in *A. thaliana* cells [13], cause extensive oxidative damage to proteins [14], increase oxidative stress defense enzyme levels in chloroplasts [14], cause dimerization of the large RuBisCO subunit [15] and induce autophagy, a housekeeping mechanism that removes damaged protein aggregates and organelles [14]. Molecular biology studies of TiO_2_ NP toxicity primarily employed transcriptomics tools. Results of transcriptomics analyses vary in relation to the identity and the number of genes that are up- or down-regulated by exposure of *A. thaliana* plants to TiO_2_ NPs [16–18]. All the studies, however, showed that there is a consistent link between TiO_2_ NP treatments and changes in the expression of genes belonging to ontology groups involved in stress responses [16–18]. So far, consistent molecular signatures of TiO_2_ NP toxicity have not been uncovered.

The variable conclusions of toxicology studies assessing the effects of TiO_2_ NPs on *A. thaliana* are likely due to the use of different growth conditions, plant growth stages, and exposure regimes [11]. Another significant variable is the choice of the type of TiO_2_ NP. TiO_2_ NPs have size-, shape- and structure-dependent physiochemical properties [1]. For example, small (≤ 20 nm) TiO_2_ NPs with an anatase crystalline structure have a specific reactivity that stems from the coordinative unsaturation of the nanoparticle surface. This energetic disbalance is compensated by binding of molecules present in the environment, and the highest affinity ligands described to date are enediols (e.g., catechols (benzene-1, 2-diols)) and other ortho-substituted bidentate compounds [19, 20]. Enediol ligands are abundant in plants, and we have previously shown that when plant tissue is incubated in a suspension of small anatase TiO_2_ NPs, these NPs are taken up by plant cells wherein they avidly gather catechol compounds on their surface. The functionalized NPs are then extruded by the cells into the incubation media [21]. This use of nanoparticles to “harvest” metabolites from living plant cells is a method for enrichment of natural products which we named “nanoharvesting” [21]. Flavonoids are a large group of polyphenolic plant natural products that have a phenylbenzopyran structure and many of them have an enediol group [22]. Our previous targeted metabolomic analyses of nanoharvesting from *A. thaliana* wild-type plants confirmed there is an enrichment of those flavonoids that have a catechol ring [21].

The flavonoid biosynthetic pathway has been analyzed in detail in many plant species [23–25]. In the plant model system *A. thaliana*, mutants affecting every step of the canonical flavonoid biosynthesis pathway have been isolated and collectively named *transparent testa* (*tt*) mutants, due to their characteristic transparent seed coats (testas) [26]. In the present study, we analyzed the composition of the bio-coronas formed on the surface of TiO_2_ NPs incubated with leaves of the wild-type *A. thaliana* and four *tt* mutants that harbor lesions in major flavonoid biosynthetic genes. By using an untargeted metabolomics approach, we aimed to determine which classes of chemicals, other than flavonoids, bind to the surface of TiO_2_ NPs and how changes in flavonoid composition alter the composition of the corona. A comparative analysis of the chemical composition of the TiO_2_ bio-corona and methanolic extracts isolated from the five selected plant lines indicates that the anatase TiO_2_ NP surface has a high affinity not only for flavonoids but also for lipids. We also show that qualitative alteration of the flavonoid profile did not alter lipid yields, but the lack of flavonoids in plant tissue led to a significant increase in the amounts of nanoharvested lipids, indicating that flavonoids and lipids compete for binding at the TiO_2_ NP surface.

## Materials and methods

### Plant material and growth conditions

The transparent testa (*tt*) mutant seeds were obtained from the Arabidopsis Biological Resource Centre (Ohio State University). The selected mutants are the ethyl methanesulfonate (EMS) mutant *tt4-1* [26] that affects the *CHS* gene (At5g13930), the fast-neutron mutant *tt5-1* [27] which carries a lesion in the *CHI* gene (At3g55120), the EMS mutant *tt7-1* [28] in which the gene encoding the branching enzyme F3′H (At5g07990) is mutated and the X-ray induced mutant *tt3-1* [27] that affects *DFR* (At5g42800). The EMS mutant *tt6-1* that maps to *F3H* (At3g51240) has not been included in our analyses as it was previously proven that the mutation does not fully abolish the function of the enzyme [29]. The seeds were surface sterilized (1 min 10% bleach, followed by three rinses with 70% ethanol and three rinses with sterile water) and sown on half-strength Murashige and Skoog medium [30] containing 5% sucrose and 0.8% agar (pH 5.7). Plants were grown in a controlled environmental chamber at 23 °C with continuous illumination for 5 weeks. All mutants are referred to without the allele specification throughout the manuscript.

### Extraction procedures

A pool of three mature leaves excised from separate plants was used for both the methanolic extraction and nanoharvesting. For methanolic extracts, leaves were frozen in liquid nitrogen and disrupted using zirconium beads and a bead beater for 1 min at 4000 rpm in 10 volumes of 1% HCl/methanol containing 0.1 mg/L lidocaine (recovery standard). The extraction was continued for 16 h in the dark at 4 °C. Samples were then centrifuged at maximum speed for 15 min at 4 °C and the supernatants were mixed with an equal volume of chloroform to remove chlorophylls. Phases were separated by centrifugation and the upper methanolic phase was used for the analyses.

For nanoharvesting, we used ultra-small anatase TiO_2_ NPs obtained from US Research Nanomaterials Inc. as a 15 wt% aqueous nanopowder dispersion (1.9 M). The anatase NPs were spherical or slightly elliptical with a size distribution between 5 and 15 nm and a composition that is 99.9% TiO_2_ (as determined by the manufacturer using transmission electron microscopy analyses and sample purity measurements [31]). As previously described [14], hydrodynamic diameter and Zeta potential measurements were done using a Malvern Zetasizer Nano ZS (HeNe laser, 663 nm; detector angle, 173) at nanoComposix (https://nanocomposix.com). Since dynamic light scattering measurements of diluted samples of very small (< 20 nm) NPs lead to inaccurate measurements [32], a concentrated nanoharvesting aqueous stock suspension (20×, 38 mM) was analyzed. The characteristics of the analyzed samples were: hydrodynamic diameter ± SD (n = 6) = 31.3 ± 17.8 nm; zeta potential ± SD (n = 6) = 39 ± 7.4 mV; the multimodal number distribution peak mean was 13.53 nm, multimodal number distribution peak width was 4.304 nm and the poly-dispersity index was 0.325. The master nanoharvesting stock (190 mM) was prepared by diluting the commercial dispersion in HPLC-grade methanol (1:9 v/v), pelleting the washed NPs (1000×*g* for 5 min), and resuspending them in HPLC-grade water. Immediately before the nanoharvesting experiment, the master stock was diluted in HPLC-grade water to a final concentration of 1.9 mM and sonicated in an ultrasonic water bath for 5 min. For each sample, three mature leaves were immersed in 1.9 mM TiO_2_ NPs suspension and incubated at 22 ºC in the dark for 4 h. The leaves were then removed and the harvested material was pelleted (1 min at 3500 rpm). For elution of compounds bound to the particle surface, 1% HCl/methanol containing 0.1 mg/L lidocaine was added to the nanoconjugate pellet and the pellets were disrupted by zirconium beads in a bead beater (2 min at 4000 rpm) followed by sonication for 2 min. After a brief spin (30 s at 3500 rpm), the nanoconjugate extract was mixed with an equal volume of chloroform. The phases were separated by centrifugation and the upper methanolic phase was used for the analyses. Prior to the LC–MS/MS analyses, all samples were filtered through 0.22 micron filters (Cameo 3N, GE Waters).

### Untargeted metabolomics analyses

Untargeted MS analysis was performed at the Proteomics & Mass Spectrometry Facility at the Danforth Plant Science Center. The LC–MS/MS platform used was Q-Exactive with 50,000 mass resolution (Thermo-Fisher Scientific) with TriVersa Nanomate (Advion) and 2DLC Ultra NanoLC (Eksigent). The samples were injected onto 0.5 × 50 mm Supleco Bioshell C4 column using 0.1% formic acid in water (A) and acetonitrile (B). The gradient was as follows: 25%B for 3 min followed by a linear ramp to 100%B over 11 min, a hold at 100%B for 5 min then a ramp back to 25%B over one minute followed by re-equilibration for 10 min. Data were collected in a polarity switching mode and MS/MS spectra were acquired in a top 5 data-dependent acquisition experiment. Data were preprocessed and viewed using the Elements software package (https://www.proteomesoftware.com). Peak assignments were made based on exact mass, isotope pattern match and MS/MS spectral match against the NIST spectral database.

### Data analyses

Preprocessed datasets were exported from Elements as peak intensity tables (*.csv) and analyzed using MetaboAnalyst 3.0 (https://www.metaboanalyst.ca) [33]. After missing value imputations and data filtering, data were normalized by sum, glog2 transformed and autoscaled. Preliminary data analyses were done using the Statistical Module of the MetaboAnalyst. For in-depth analyses, the normalized data were analyzed using the R package *mseapca* [34]. Data were scaled to zero mean and PC scores, factor loadings and *p*-value and *q*-value by Benjamini and Hochberg were calculated. Metabolites with significant factor loadings were sorted into chemical classes or subclasses following the Human Metabolome Database (HMDB, https://www.hmdb.ca/) classification. Hierarchical clustering analysis (HCA) was done using the R package *ComplexHeatmap* [35] using Manhattan clustering distance and ward.D2 clustering method.

### Cell viability analyses and confocal microscopy

Wild-type seeds were surface sterilized and plated on half-strength Murashige and Skoog media. Four-day-old seedlings were incubated in water or 1.9 mM aqueous suspension of TiO_2_ NPs for 4 h in the dark. For viability analysis, seedlings were stained with SYTOX green as previously described [36]. Images were acquired on an Olympus Fluoview 1200 confocal microscope and Z-stacking was performed using the Flouview software.

## Results and discussion

### Methanolic and nanoharvesting extracts

We have previously shown that TiO_2_ NPs are taken up by plant cells, wherein they become coated with flavonoids and from which they get extruded back into the nanoharvesting suspension as flavonoid-NP conjugates [12, 13, 21]. To test if any other classes of metabolites aside from flavonoids bind to TiO_2_ NPs in the plant cell, we grew wild-type and *tt* mutant plants on sterile media and incubated the leaves in an aqueous TiO_2_ NP suspension. After a 4-h-long incubation in the dark at room temperature, leaves were removed, the coated nanoparticles were pelleted, and the bound metabolites were freed from the particle surface using acid methanol. These acid–methanol extracts are referred to as nanoconjugate eluates (NEs). In parallel, leaves were incubated with acid methanol to obtain the control extracts (methanolic extracts, MEs) that should contain all leaf metabolites that are soluble under these conditions.

**Fig. 1:**
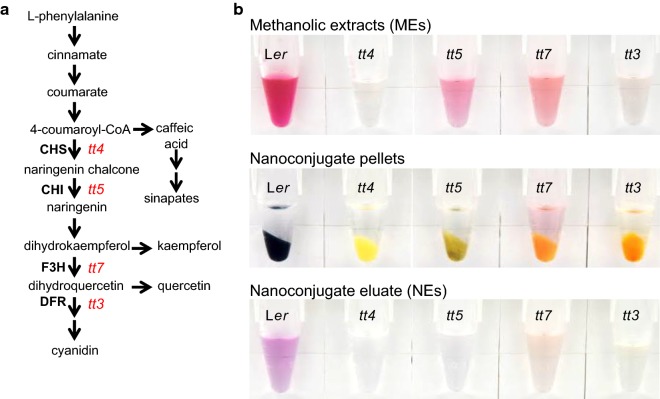
Preparation of methanolic (MEs) and nanoparticle-mediated extracts (NEs) from wild type and *tt* mutant lines. **a** Simplified scheme of the phenylpropanoid pathway in Arabidopsis. The positions of the *tt* mutations used in this study are marked in red and the abbreviated names of the enzymes compromised are boldfaced. CHS, Chalcone synthase; CHI, chalcone isomerase; F3H, flavanone 3-hydroxylase; DFR, dihydroflavonol reductase. **b** Visual characteristics of MEs, nanoconjugates and NEs from different plant lines

Considering the genetic lesions in the *tt* mutants (Fig. 1a), the *tt4* MEs should have no flavonoids, the *tt5* MEs should not contain any flavonol (kaempferol and quercetin, yellow) or anthocyanin derivatives (red and purple), the *tt7* MEs should have no quercetin or anthocyanin derivatives, and *tt3* MEs should have no anthocyanins [29]. Surprisingly, the MEs of *tt5* and *tt7* plants were visibly purple, suggesting that anthocyanins are present and that these mutations are either leaky or that the affected genes were functionally complemented by others (Fig. 1b). Previously, cold-acclimated *tt5* and *tt6* plants were shown to contain flavonols and anthocyanins, albeit at a lower level than the extracts of the cold-acclimated wild-type [37]. All plants used in our study were grown on high-sucrose (5%) Murashige and Skoog medium, a growth condition that is know to increase the amount and diversity of flavonoid species [21, 38–40]. High sucrose content of the growth media is perceived as environmental stress and leads to an up-regulation of flavonoid biosynthetic genes through the action of the MYB75/PAP1 transcription factor [39, 41]. Collectively, these results suggest that under stress conditions *tt5, tt6* and *tt7* mutant plants recruit alternative enzymes to the flavonoid biosynthetic pathway. Irrespective of the underlying reason for the accumulation of anthocyanins in *tt5* and *tt7* plants, we opted to exclude these two mutant lines from further analyses as their unexpected stress-induced flavonoid response added additional complexity to the study.

The conjugation of compounds to the surface of anatase TiO_2_ NPs is known to lead to a shift in resonant properties of the nanoparticles which can be measured as a change of the absorbance maxima in the UV/Vis spectrum or observed by eye if the nanoconjugates absorb in the visible light range [42]. In the case of flavonoids, binding of purple anthocyanins leads to the formation of blue nanoconjugates, and binding of pale yellow flavanols such as quercetin leads to the formation of orange nanoconjugates [21]. Indeed, the nanoconjugates isolated from wild-type plants, which are rich in purple anthocyanins, were blue and nanoconjugates from mutants that have no anthocyanins at all (*tt4* and *tt3*) were yellow or orange (Fig. 1b).

### Comparisons of the MEs and NEs isolated from individual lines

**Fig. 2:**
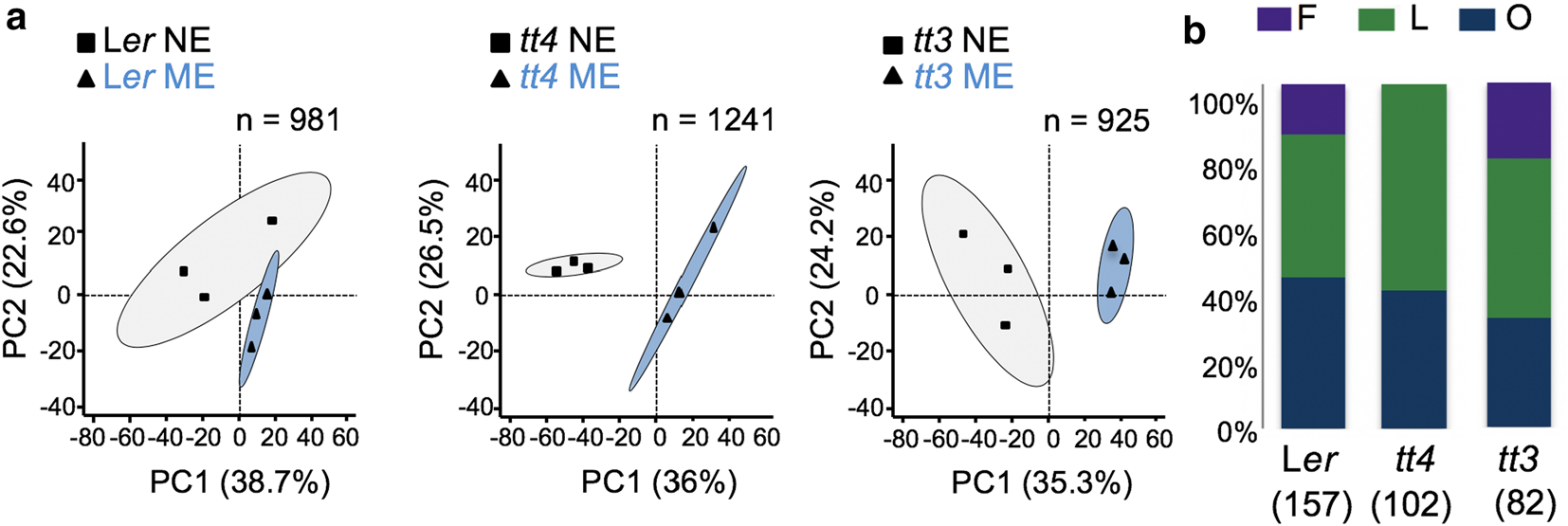
Comparison of methanolic (MEs) with nanoconjugate (NEs) extracts from individual lines. **a** PCA of untargeted metabolomics data demonstrating the separation of MEs and NEs from the wild type L*er,* and from the mutants *tt3* and *tt4*. Principle component (PC) 1 and PC2 are presented. Three biological replicates were used for these analyses. The number of metabolites analyzed (n) is noted. **b** Distribution of metabolites with significant loading in PC1 into chemical classes. The number below the plant line identifier denotes the number of endogenous metabolites with significant loading that were sorted into chemical classes. F, Flavonoids; L, Lipids; O, Other

**Fig. 3:**
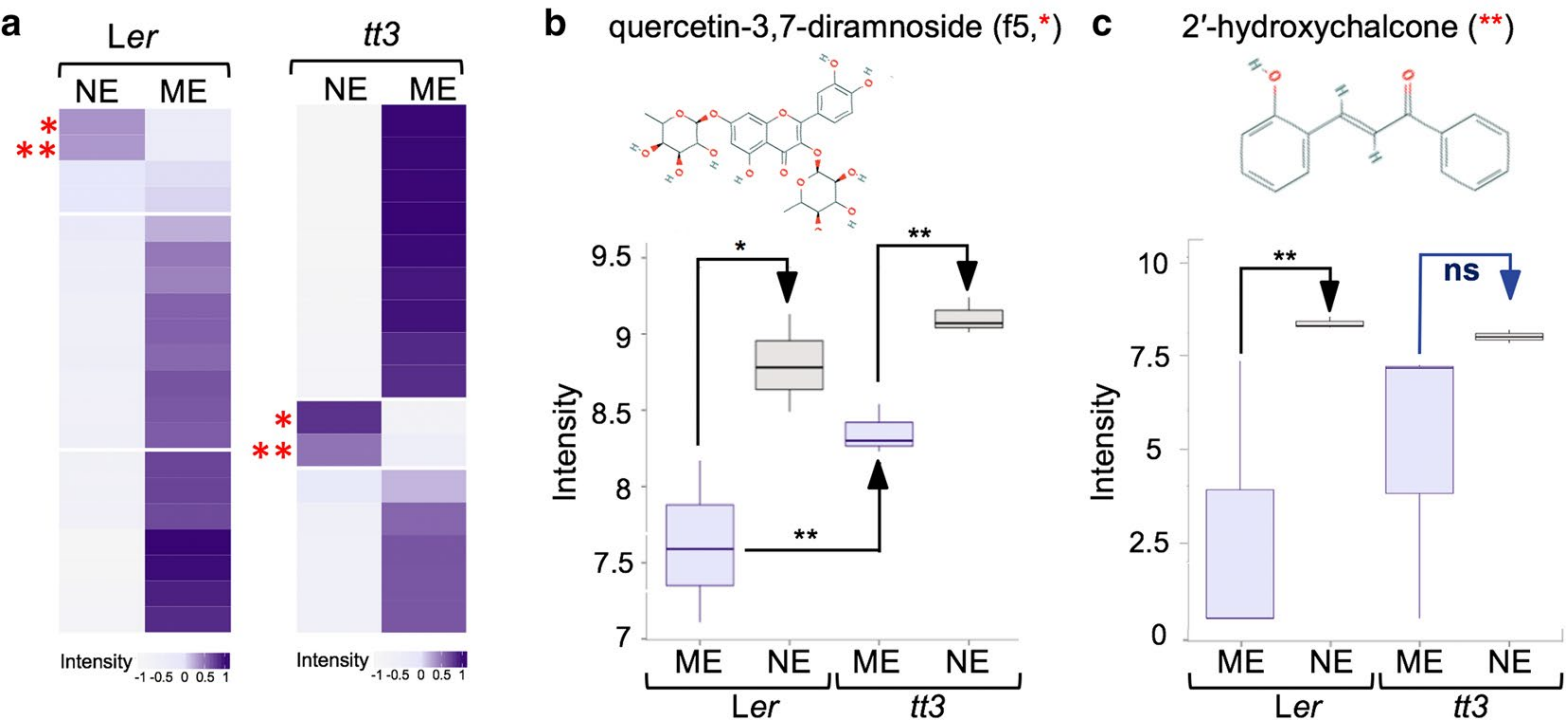
Comparative analyses of flavonoids in extracts from the wild type and the *tt3* mutant. **a** The heatmaps were constructed from the average normalized intensity value of flavonoids extracted by the PCA analyses from the full data set. Hierarchical clustering analysis was performed on 20 (L*er*) and 16 (*tt3*) metabolites with significant factor loadings in PC1 (p = 0.05) using Manhattan correlation as the distance measure. The color intensity scale is positioned below the heatmap. Starts denote: *, quercetin-3,7-dirhamnoside (f5) and **, 2′-hydroxychalcone. NE, nanoconjugate eluate; ME, methanolic extract. **b**, **c** Box plots of log10 metabolite intensity of f5 (**b**) and 2ʹ-hydroxychalcone (**c**) in the wild type (L*er*) and *tt3* extracts. The significance between means (n = 3) was calculated using Students *t*-test. Error bars represent mean standard deviation. *p < 0.05, **p < 0.005, NS, not significant

**Fig. 4:**
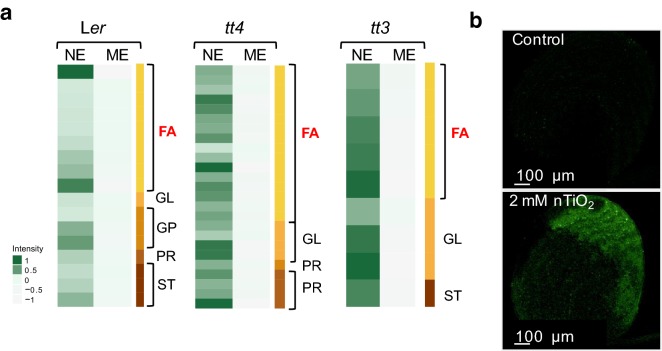
Comparative analyses of lipids in extracts from the wild type and *tt* mutants. **a** The heatmaps were constructed from the average normalized intensity values of lipids shown to be significantly different between NEs and MEs by PCA analyses. The color intensity scale is on the left-hand side of the heatmaps. Different lipid subclasses are labeled with different colors in the legend positioned on the right-hand side. FA, fatty acyls; GL, glycerolipids; GP, glycerophospholipids; SP, sphingolipids; ST, sterol lipids; PR, prenol lipids. **b** Confocal microscopic analysis of TiO_2_ NP-treated wild-type seedlings reveals membrane damage. Z-stack projections (10 slices, 25 µM thick) of SYTOX green-stained cotyledon of a 4-day-old seedling treated with 1.9 mM TiO_2_ NP for 4 h is shown. Green fluorescence signifies membrane permeabilization that allows the dye to enter the cell, bind DNA and emit at 510–560 nm when excited by a wavelength of 488 nm

**Fig. 5:**
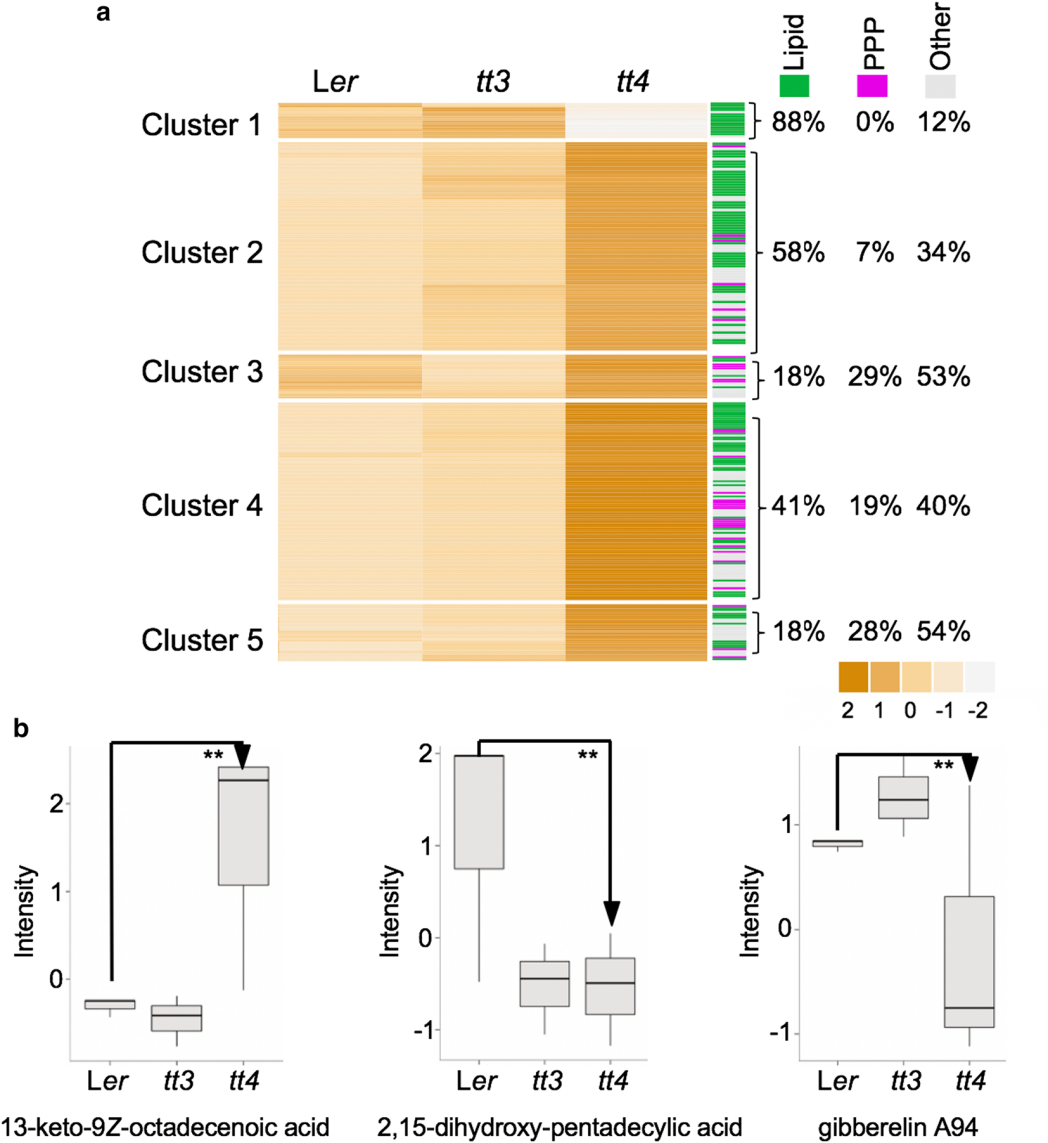
Comparative analyses of NEs from the wild type and *tt* mutants. **a** Each of the heatmap columns represents the normalized intensity averaged across the NE samples within each group (n = 3, each sample was a pool of tissues from separate plants). Hierarchical clustering analysis was performed on metabolites with significant factor loadings in PC1 (p = 0.005) using Manhattan correlation as the distance measure. Percentage of lipids (green), phenylpropanoids (PPP; purple) and other compounds (gray) that form each cluster is noted on the right-hand side. The color intensity scale is positioned below the heatmap. **b** Boxplot analyses of ME levels of select lipids that have been enriched in the NEs of *tt4*. The significance between means (n = 3) was calculated using Students *t*-test. **p < 0.01

Previous targeted metabolomics analyses have shown that incubation of anatase TiO_2_ NPs with a complex mixture of plant compounds leads to the selective enrichment of specific flavonoids [21]. To analyze whether any other classes of compounds are selectively bound to TiO_2_ NPs exposed to the whole-leaf metabolome, we first compared the NEs with control MEs of each line to obtain an overall view of metabolite classes that have affinity for the NP surface (Figs. 2, 3, 4) and then compared the NEs of all lines (Fig. 5).

The untargeted metabolomics data were first processed and then analyzed by principal component analyses (PCA), which revealed an extraction-method dependent clustering within lines (Fig. 2a). Statistical hypothesis testing for factor loading in PC1 was performed using *mseapca* R package, and metabolites that were isolated with different efficacy (p < 0.05) by methanolic extraction compared to NP surface-dependent extraction were selected for further analyses. Based on their chemical structures, these metabolites represented three classes: flavonoids, lipids, and “others” which included ~ 25% metabolites that belong to the phenylpropanoid pathway (Fig. 2b). The distribution of classes was specific for each line (Fig. 2b). We proceeded by analyzing these three classes separately.

Previous targeted metabolomics analyses of the Col-0 wild-type revealed that specific flavonoid species are enriched in NEs [21]. Therefore, as quality control for the untargeted metabolomics data obtained in the present study, we first compared the ME with the NE of the L*er* wild type and the MEs with the NEs of the different *tt* mutants. Hierarchical cluster analyses showed that, as expected, there were no flavonoids in the *tt4* mutant (thus, not shown). Two compounds were specifically enriched in the NEs of both the wild-type and the anthocyanin-less but flavonoid-containing *tt3* mutant (Fig. 3a). The first flavonoid species enriched in both NEs was identified as quercetin-3,7-dirhamnoside, which is also known as f5 [43]. There was a significant increase in f5 level in MEs of the *tt3* mutant (Fig. 3b), which is expected as the activity of DFR is compromised in this mutant, resulting in the accumulation of quercetin and its derivatives (Fig. 1a). The enrichment of f5 in both NEs confirms the results of our previous targeted analyses of flavonoids that bind with high efficiency to the surface of anatase TiO_2_ NPs [21] and serves as a positive control that validates further analyses of the dataset. The second enriched compound was identified as 2ʹ-hydroxychalcone, suggesting that this chalcone derivative is also a high-affinity target for TiO_2_ NPs (Fig. 3c).

Statistical hypothesis testing for factor loading in PC1 showed that from the compounds that are enriched by nanoharvesting, 42% (for L*er*), 60% (*tt4*) and 46% (*tt3*) were lipids (Fig. 4a). Interestingly, fatty acids comprised 16%, 41% and 24% of the lipids differentially isolated by MEs and NEs from L*er*, *tt4* and *tt3*, respectively. Thus lipids, and in particular fatty acids, are high-affinity in vivo ligands for TiO_2_ NPs. Since the percentage of total lipids and the percentage of fatty acids were the highest in NEs of *tt4*, which contains no flavonoids, we concluded that there is competition for NP surface binding between flavonoids and lipids. These findings imply that TiO_2_ NPs can be used both to reduce or deplete lipids and in particular, the fatty acids from a complex mixture of compounds (e.g., for cleanup of water polluted with fatty acids which are well-known foaming agents) and to enrich an extract for specific lipids.

The abundance of lipids, and in particular polar membrane-associated lipids, in methanolic extracts is expected, but the finding that lipids are abundantly bound to the nanoparticle surface was surprising. Nanoparticles have been shown to be actively taken up by plant cells via the endocytotic pathway [44, 45], suggesting that the nanoparticle bio-corona is formed by NP interaction with metabolites within the cell. However, TiO_2_ NPs are known to generate reactive oxygen species and cause oxidative damage in the cell [6, 12]. Thus, it is possible that during the co-incubation of leaves with TiO_2_ NPs, plasma membranes are damaged and a fraction of nanoharvested metabolites are bound to the nanoparticles extracellularly in the co-incubation solution. To test that, we performed cell viability assays (Fig. 4b). These assays showed that co-incubation of leaves with TiO_2_ NPs compromises the integrity of the plasma membrane (Fig. 4b). Thus, it is likely that the compounds bound to the surface of TiO_2_ NPs are a mixture of molecules released from different cellular compartments due to the NP-induced cellular damage, and molecules that were bound in the cell to which NPs were delivered by endocytosis. These findings also imply that decreasing membrane damage by, for example, functionalization of the NP surface with bidentate ligand antioxidants such as ascorbic acid could improve the nanoharvesting of intracellular compounds.

### Comparison of NEs

Next, we compared the NEs from the wild-type, *tt3* and *tt4*. Hierarchical clustering analysis was done on 248 NE metabolites that had a PC1 factor loading significance of p < 0.005 (Fig. 5a). These analyses showed that the *tt4* NE was significantly different from the wild-type and the *tt3* mutant (Fig. 5a). Out of five clearly defined clusters, only Cluster 1 grouped metabolites (17 in total) that were less abundant in *tt4* NEs compared to L*er* and *tt3*, and the majority (15) of these metabolites were identified as lipids. The other four clusters contain metabolites that were enriched in *tt4* NEs. For Cluster 2 (37 metabolites) and Cluster 4 (102 metabolites), 58% and 41% of the compounds were identified as lipids with fatty acids being the most abundant group (Fig. 5a). Cluster 3 (12 metabolites) and Cluster 5 (15 metabolites) contained compounds that are not lipids or phenylpropanoids (Fig. 5a).

One possible cause for the enrichment of lipids in *tt4* is that under our growth conditions, the *tt4* mutant has a different lipid composition compared to the other plant lines. To test that hypothesis, we analyzed the ME levels of three lipids that were highly enriched in *tt4* NEs: 2,15-dihydroxypentadecanoic acid, (*Z*)-13-oxooctadec-9-enoic acid and gibberellin A94. Except for (*Z*)-13-oxooctadec-9-enoic acid, which was indeed more abundant in *tt4,* the other selected lipids were less abundant in *tt4* MEs compared to the wild-type MEs (Fig. 5b). This suggests that an absence of high-affinity targets (i.e., flavonoids) for TiO_2_ NPs in the *tt4* mutant is a more decisive factor for NP-dependent lipid enrichment than their overall abundance in the cell.

## Conclusions

Entry of NPs into an environment leads to the absorption of reactive molecules present in that environment onto the NP surface. If NPs enter a biological system, the adsorbed molecules form a bio-corona, which is a dynamic system that can include proteins, peptides, lipids, nucleic acids, and different metabolites [46]. The composition of the bio-corona is primarily dependent on the physicochemical properties of the NPs, the chemical composition of the nano-biointerface, and the duration of incubation [47]. The formation of a bio-corona leads to changes in both the physicochemical characteristics of the NPs (and thus NP reactivity, fate, and toxicity) and the biochemical composition of the “hosting” organism.

In this study, we show that although the bio-corona of ultra-small anatase TiO_2_ NPs is composed of a complex mixture of metabolites, there is a significant enrichment for flavonoids and lipids with flavonoids being the preferred binding partners. The first implication of this finding relates to the nanoharvesting methodology itself: modification of NPs and the selection of plant growth conditions or varieties (to enrich the metabolome for natural products of interest) could improve nanoharvesting efficiency and specificity. For example, coating the NPs with lipids prior to nanoharvesting could enhance binding selectivity by shielding the NP surface from low-affinity binding partners.

The second implication of our findings relates to specific concerns about the biological effects of the binding of specific classes of metabolites to the TiO_2_ NPs surface. TiO_2_ NP preparations, many of which include small anatase NPs, are abundantly used, and as a result, these NPs have accumulated in the environment causing increased concerns about their impact on different species [42, 48, 49]. Here, we need to make a distinction between two potential consequences of the sequestration of flavonoids and lipids: effects of NPs present in the soil on the plant ecosphere and in particular the rhizosphere, and effects of NPs taken up from the soil into plant cells. Irrespective of the biosystem analyzed, the main mechanism of toxicity of all nanomaterials is the generation of reactive oxygen species [50]. However, it has been shown that nanomaterials can also cause cytotoxicity by mechanisms that are not linked to the generation of free radicals (e.g., [51]). Our results suggest that alterations in lipid signaling and the effects of qualitative and quantitative changes in flavonoid composition are two additional mechanisms by which TiO_2_ NPs may affect biological functions. The internalized NPs may disrupt processes that involve lipid signaling (e.g., plant hormonal responses and responses to abiotic stresses [52]). In contrast to mammalian cells [53], intracellular flavonoid signaling in plant cells has remained largely unexplored. The antioxidative function of flavonoids, on the other hand, has been investigated and confirmed in plant systems [54–56]. Preferential binding and sequestration of flavonoid antioxidants to the surface of reactive oxygen species-generating NPs may significantly reduce the antioxidative capacity of plant cells and thus, increase their susceptibility to environmental stresses and disbalance the redox signaling mechanisms which are required for plant stress defense responses [57].

Contrary to intracellular flavonoid signaling, the importance of flavonoid signaling in the rhizosphere has been extensively investigated. Flavonoids are abundant in root exudates and have been shown to cause chemoattraction of nitrogen-fixing bacteria of the genus *Rhizobium* towards the roots of Legumes and regulate the expression of rhizobial *nod* genes [58–60]. Secreted flavonoids also inhibit root pathogens and mediate allelopathic interactions between plants [61]. Adsorption of flavonoids to the surface of TiO_2_ NPs present in the rhizosphere could interfere with all these flavonoid-dependent processes. In the case of the rhizobia-legume symbiosis, this would disrupt a process that many agricultural producers rely on to reduce the need for nitrogen fertilizer applications. Interestingly, delayed or decreased nodulation has been reported in two legume-rhizobium systems challenged with TiO_2_ NPs [62, 63]. In another study of the effects of TiO_2_ NPs on symbiotic plant relationships, TiO_2_ NPs were shown to have an inhibitory effect on arbuscular mycorrhizal symbiosis in plant roots [64]. Since flavonoids have been shown to increase spore germination for several arbuscular mycorrhizal fungal species [65], we can hypothesize that adsorption of flavonoids to TiO_2_ NPs could play a role in their inhibition of also this symbiotic relationship.

In summary, a better understanding of how TiO_2_ NPs interacts with organic molecules will help the assessment of their potential environmental impacts and the development of tools to counteract the adverse environmental effects of this ubiquitously utilized nanomaterial.

## Data Availability

The datasets analysed in the current study are available from the corresponding author on reasonable request.
